# Size specific dose estimate (SSDE) for estimating patient dose from CT used in myocardial perfusion SPECT/CT

**DOI:** 10.22038/aojnmb.2019.40863.1276

**Published:** 2020

**Authors:** Vishnukumar Rajaraman, Madhusudhanan Ponnusamy, Dhanapathi Halanaik

**Affiliations:** Department of Nuclear Medicine, Jawaharlal Institute of Postgraduate Medical Education and Research, Puducherry, India

**Keywords:** SSDE, CTDI, Patient dose in CT

## Abstract

**Objective(s):**

Size specific dose estimate (SSDE) is a new parameter that includes patient size factor in its calculation. Recent studies have produced mixed results on the utility of SSDE, especially when automatic exposure control (AEC) was used. The objective of the study was to find out if there is a relationship between patient size and each of the parameters, SSDE and CTDIvol, when AEC is used.

**Methods:**

CT data of consecutively selected 111 patients were included for analysis. CTDIvol values of the CT scans were extracted for each patient. Effective diameter of each patient was calculated as geometric mean of anteroposterior and lateral diameters measured on axial CT images. Corresponding conversion factors for effective diameters were obtained from American Association of Physicists in Medicine (AAPM) report 204. SSDE was obtained as the product of CTDIvol and conversion factor values. Linear regression model was used to evaluate the relationship between patient size and the parameters SSDE and CTDIvol.

**Results:**

Mean weight was 62 (11.5) and range was 34 - 103 kg. Median CTDIvol (mGy) on AEC mode was 7.27(IQ range 7.27, 7.65) and mean effective diameter was 26.2 cm (2.4). Mean SSDE (mGy) was 10.6 (0.84). Good positive correlation was obtained between CTDIvol and effective diameter (r=0.536; p<0.0005). Strong inverse correlation was noted between SSDE and effective diameter (r=-0.777; p<0.0005). Linear regression model for establishing relationship between CTDIvol and effective diameter showed slope of 0.314mGy/cm (R=0.561; R2=0.314; P<0.0005) whereas between effective diameter and SSDE slope was -0.23mGy/cm (R=0.676; R2=0.457; P< 0.0005).

**Conclusion:**

The study shows that CTDIvol and SSDE vary but divergently, with patient size. SSDE is a better estimate of patient radiation dose from CT of MPI SPECT/CT than CTDIvol in systems that use automated exposure control.

## Introduction

 Computed tomography (CT) plays an integral part in the diagnosis and follows up of several disease conditions. CT is widely used in hybrid imaging for diagnostic as well as for attenuation correction purposes. All medical exposures should be justified, as low as reasonably achievable (ALARA) and should not exceed the individual dose limitations (1). Current radiation protection approaches are modelled based on the assertion that every radiation dose of any magnitude will produce some detrimental biological effects. Therefore, it is reasonable to report patient dose from any radiological investigation using ionising radiation. 

 CT-based attenuation correction is usually employed in myocardial perfusion imaging in single photon emission computed tomography (SPECT) and positron emission tomography (PET). CT delivers additional radiation dose to the patient. Previous systems used non-CT based attenuation correction methods like use of a radioactive source to produce transmission scans. Cost, poor count density, and maintenance issues limit the wide usage of gamma sources for attenuation correction while CT-based correction is more convenient. Cardiac specific gamma cameras with solid state detectors provide improved spatial resolution and detection efficiency and hence avoid the need for attenuation correction. However, high equipment cost has led to limited usage. Therefore, hybrid SPECT/CT systems have become popular for myocardial perfusion imaging. Various parameters were used for estimating patient dose from CT, the commonly used parameters being volumetric CT dose index (CTDIvol) and Dose length product (DLP).

 CT dose index (CTDI) integrates radiation dose profile of a single scan along Z-axis and normalized to the slice thickness. To standardise for scan length (Z-axis), 100 mm ionisation chamber was used to measure CTDI100. But CTDI100 varied with different body fields. This limitation of CTDI100 is overcome by the use of weighted CTDI (CTDIw), which sums 1/3rd of dose from the centre of the phantom and 2/3^rd^ of dose from periphery of the phantom. CTDIw could be used in scans only when there was no table movement (2).Volumetric CTDI (CTDIvol) is used for spiral CT scanners. It measures scanner output and not patient dose. Studies showed that the measured patient dose was different from the displayed dose of CTDIvol (3-5). 

 To address this variation, the American association of physicists in medicine (AAPM) formed a task group to develop an appropriate tool for estimating patient dose from CT scan. In their report number 204, they recommended size specific dose estimate (SSDE) for estimating patient dose (6). The group suggested use of conversion factors based on effective diameter (ED), lateral diameter (LD) and anteroposterior diameter (APD). These factors differ based on the size of the phantom used; the most common sizes being 16 cm and 32 cm. SSDE is calculated as the product of CTDIvol displayed on the scanner and conversion factor. 

 Some studies showed that SSDE depends on patient size. CTDIvol and SSDE values differed for patient weight ranging between 36 and 100 kg. CTDI32vol correlated with SSDE for patients weighing between 100 and 140 kg, CTDI16vol correlated with SSDE for patients weighing less than 36 kg. In patients ranging between 36 and 100 Kg CTDIvol did not give proper estimate and require more realistic dose estimates like SSDE (7).

 Automatic exposure control (AEC) technology allows adjustment of tube current and time product based on patient body characteristics. In scanners with AEC technology, the scanner-provided CTDIvol values are dependent on patient size. Studies that have used these scanners have reported contradicting results on the use of appropriate parameter for patient absorbed dose estimate. The aim of the study was to determine the relationship of SSDE and CTDIvol with patient size.

## Methods

 After obtaining approval from the Institute Ethics Committee, myocardial perfusion SPECT/CT data of 111 consecutive patients who underwent the scan between November 2017 and January 2018 were analysed. Standard procedure was followed for myocardial perfusion SPECT/CT on Siemens Symbia T6 dual headed gamma camera incorporated with CARE 4D software for CT acquisition. The CT acquisition parameters were 130 kVp, pitch factor of 0.4 and slice thickness of 5 mm. Scan length was fixed between aortic arch and costodiaphragmatic recess. CT was always performed prior to SPECT.

 The following information was obtained from de-identified scans as displayed by the system: CTDIvol, DLP, mAs, and kVp. Anteroposterior diameter (APD) and Lateral diameter (LD) were manually measured on the CT images using digital callipers. The mid slice that showed maximum diameter of heart was chosen for this purpose. Effective diameter was calculated using the following formula. 

Effective diameter=√ (APdiameter×lateraldiameter)

 AAPM conversion factors based on effective diameter for CTDI_vol_32 (6). Finally, SSDE was calculated for each patient as the product of conversion factor and CTDI_vol_32. After checking the normalcy of data, Spearman correlation was used for determining the correlation between SSDE, CTDIvol and effective diameter. Linear regression analysis was used to find out the relationship between SSDE, CTDIvol and effective diameter. Continuous variables were expressed in mean (SD). Data was analysed using SPSS version 21.0.

## Results

 Total participants in our study were 111 patients, which included 79 males and 32 females. Mean age was 54.2 years (12.6) ranged between 21 and 85 years. Mean weight was 62 kg (11.5) ranged between 34 and 103 kg. Median mAs was 71 (71-105). Mean AP diameter, lateral diameter and effective diameter (cm) were 21.6 (2.1), 31.9 (3.3) and 26.2 (2.4) respectively. Median CTDIvol (mGy) was 7.5(0.57). Mean conversion factor was 1.41 (0.13) with a range of (1.10-1.84).Mean SSDE (mGy) was 10.6 (0.84). Mean difference between SSDE and CTDIvol was 3.09 (0.9) (p< 0.0005).

**Figure 1:**
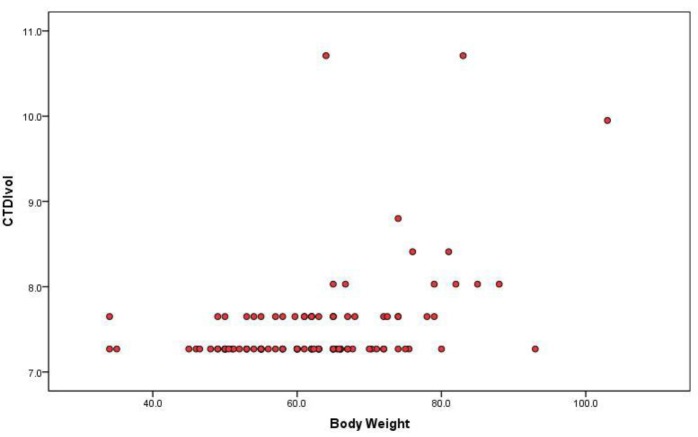
Scatter Plot showing relationship between CTDIvol and Patient’s body weight

**Figure 2:**
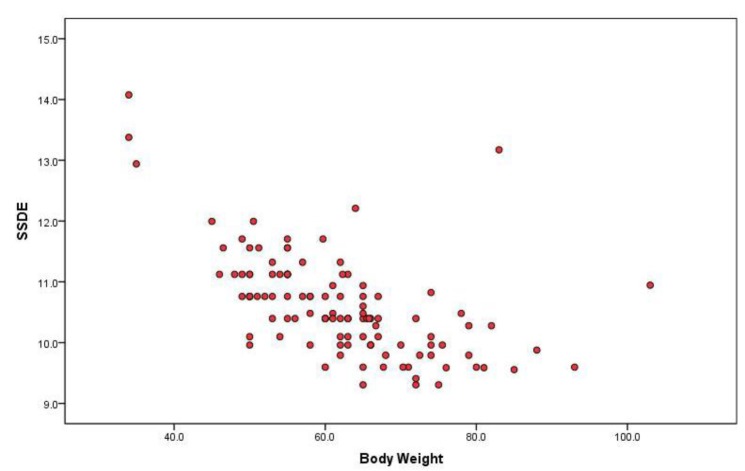
Scatter Plot showing relationship between SSDE and Patient’s body weight

 Linear regression model for establishing relationship between body weight and CTDIvol showed slope of 0.022 mGy/kg (R=0.438; R2=0.192; P<0.0005). Body weight was responsible for 19% of variations in CTDIvol. Slope of -0.042mGy/kg was obtained in linear regression model for SSDE and body weight (R=0.578; R2=0.334; P< 0.0005); body weight was responsible for 33% of variations in SSDE. SSDE values decreased with increase in body weight (Figure 2). CTDIvol showed weak positive correlation with patient weight. CTDIvol values did not vary much with body weight of the patient (Figure 1).

**Figure 3:**
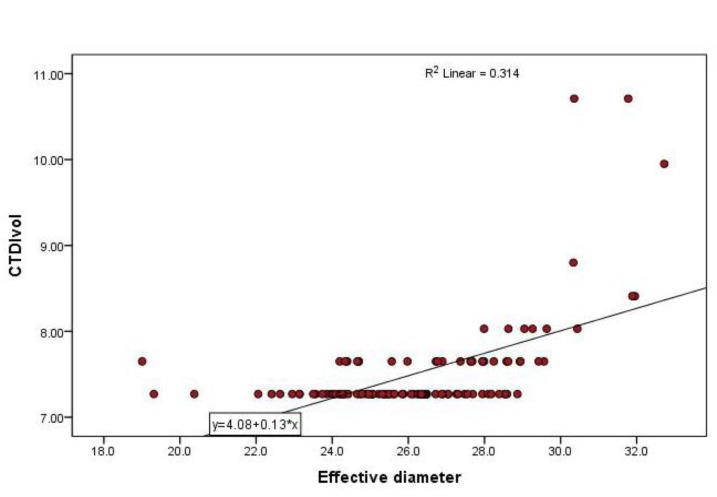
Scatter Plot showing positive relationship between CTDIvol and Patient’s effective diameter

**Figure 4:**
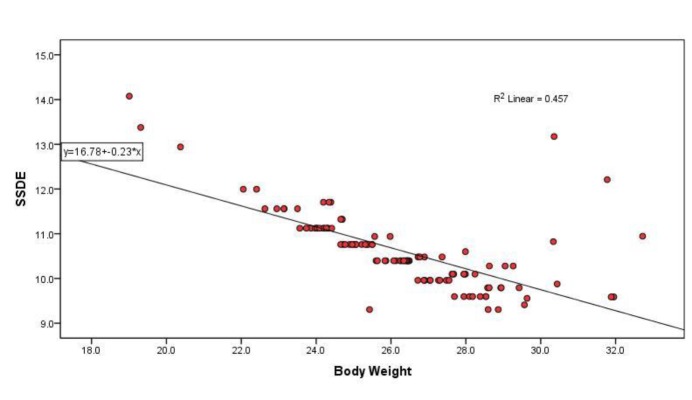
Scatter Plot showing inverse relationship between SSDE and Patient’s effective diameter

 Spearman’s correlation between CTDIvol and effective diameter showed moderate linear positive correlation (r=0.536; p <0.0005). Strong linear negative correlation was noted between SSDE and effective diameter (r= -0.777; p <0.0005). Linear regression model for establishing relationship between effective diameter and SSDE showed slope of-0.23mGy/cm (R=0.676; R2=0.457; P < 0.0005), whereas for CTDIvol and effective diameter slope was 0.13mGy/cm (R=0.561; R2=0.314; P<0.0005). Effective diameter (patient size) resulted in 46% of variation in SSDE and 31% of variation in CTDIvol(Figure 4 and 3 respectively). Almost for all CTDIvol values, the corresponding SSDE values were high. This indicates that CTDIvol might have underestimated patient dose for the particular patient size selected for analysis.

## Discussion

 In our sample, CTDIvol values were found to be lower than SSDE values. The mean weight of our study population was 62 kg (11.5). A previous study by Brady and Kaufmann showed similar results (7). They had analysed 186 patients who underwent chest, abdominal and pelvic CT examinations. Mean weight in their study group was 37.7 kg (33.1). CTDIvol16 values were in agreement with SSDE values in patients weighing less than 36 kg; similarly CTDIvol32 values were in agreement with SSDE values in patients weighing between 100 kg and 140 kg. However, SSDE values were 1.2 to 1.5 times greater than CTDIvol32 values in patients weighing between 48 and 77 kg. The SSDE values found in our group of patients were 1.4 times greater than CTDIvol32 values. This suggests that the use of CTDIvol32 in patients weighing 48 and 77 kg may not be appropriate to estimate patient dose. In our study, most of the patients weighed between 50 and 70kg. Most of the patients in our study group belong to lower or middle socioeconomic status and the median weight of the patients is 62 kg (IQ range- 54, 67 kg). Only 6 patients out of 111 weighed less than 48 kg. Similarly, 10 out of 111 patients weighed more than 78 kg. So, subsequent relationship between SSDE and CTDIvol32 could not be assessed in patients weighing less than 48 kg and patients weighing more than 78 kg.

 Bhatt et al conducted a study in 30 lung cancer patients who had undergone CECT of thorax (8). Mean effective diameter and weight of the patients were 12.5 cm (0.9) and 58.03 kg (9.4) respectively. Conversion factors in their study group ranged between 1.91 and 1.23. Mean CTDIvol and SSDE were 270 mGy (1.6) and 388 mGy (81). CTDIvol was found to be less than SSDE. In our study, mean weight and ED were 62 kg (11.5) and 26.2 cm (2.4) respectively. Our conversion factors ranged between 1.10 and 1.84. Patient characteristics in the two study groups are comparable. They used CTDIvol that was normalized to 32 cm. These results suggest that CTDIvol32 can be less than SSDE in patients weighing 36 to 100 kg.

 Christner et al analysed 545 patients who underwent CT examinations of torso. Conversion factors ranged between 1.74 - 0.80. Mean CTDIvol and SSDE in their study were 18.1 (3.7) and 21.8 (3.4) respectively (9). In our study, conversion factors ranged between 1.10 and 1.84. Mean CTDIvol32 and SSDE values were 7.5 mGy (0.57) and 10.6 mGy (0.84) respectively. Patient characteristics of the two study groups were similar, but CTDIvol and SSDE values were different. This is probably because Christner et al included thoracic & abdomen CT examinations in their study whereas our study included thorax-only CT examinations. Since the acquisition parameters were different between the studies, differences in mean CTDIvol and SSDE values are expected. CTDIvol values in both the studies were less than SSDE values. A vast majority of the 545 patients had different CTDIvol and SSDE values.

 Tsujiguchi et al compared values of CTDIvol and SSDE in their study population of 753 patients. They found that both the parameters correlated with patient size as measured by effective diameter (10). For most of the cases, CTDIvol values were found to be less than SSDE. This indicated that CTDIvol underestimated patient dose. Ratio of SSDE to CTDIvol decreased with increase in effective diameter. Ratio indirectly provides information about the conversion factors. Conversion factors, and therefore SSDE decreased with increase in patient size. Our study displays comparable results with the above mentioned study.

 CTDIvol is only an estimate of patient dose and not an accurate method to represent patient absorbed dose. CTDI estimated patient dose by integrating dose profile from the central slice. If the tails of the dose profile are not included, measured dose can be less than the actual dose. CTDIvol was calculated based on the results of experiments with 16 and 32 cm (Poly (methyl methacrylate)) PMMA phantoms. Radiation doses are high at the surface and gradually decreases as it moves towards the centre of the phantom. For adults, CTDIvol are calculated using 32 cm phantom. For that particular size, dose contributed by the central slice is less compared to dose that is received by a patient whose body diameter is less than 32 cm (11). We used absorbed dose values derived from a 32 cm phantom and therefore, these values are less likely to be appropriate for our study patient population who were considerably small-sized. Therefore, CTDIvol32 underestimates absorbed dose.

 We found positive correlation between CTDIvol and patient size (represented by body weight and effective diameter) and negative correlation between SSDE and patient size. CTDIvol showed moderate correlation with patient size (R2= 0.314; P< 0.0005).CTDIvol is a measure of scanner output. Scanner output varies with mAs and kVp. By altering the tube current, scanner output varies and CTDIvol values also vary in response to it. Therefore in scanners with automatic exposure control, tube current is modulated based on APD and LD. Since the CT scanner used by us has the capability for tube current modulation, CTDIvol values are expected to vary with patient size. Therefore, CTDIvol will be dependent on patient body habitus, even if they have same effective diameter. SSDE in these cases might also vary - first, because of different conversion factors, and secondly due to different CTDIvol.

 Nickoloff et al studied impact of scan parameters on different sizes of phantoms (12). They used different phantom sizes ranging from 6 cm to 32 cm. They compared the variation of tube current with phantom diameter. Measure of scanner CTDIvol varied exponentially with scan parameters. If the scan parameters were kept constant, absorbed dose decreased with increase in the size of the phantom. This supports two of the important findings in our study. First, it supports the variation of CTDIvol with patient size. Second, it supports the negative relationship of patient dose measured by SSDE with patient size. In tube current modulation systems, the target mA and kVp will be fixed after topogram or scout images and kept constant for each scanner rotation.

 Therefore when scan parameters are kept constant, the absorbed decreases with increase in patient size and SSDE decreases with increase in patient size.

 In a study on 509 patients who underwent myocardial perfusion SPECT/CT, Abdollahi et al found that the mean CTDIvol was 1.34 mGy (SD 0.19) and the mean SSDE was 1.7 mGy (SD 0.16). CTDIvol was strongly dependent on patient size, but SSDE was not (13). Regression model showed that patient size had a 0.025 mGy/cm slope and R2=0.505 (95% CI: P<0.0001). Linear regression model for SSDE showed (95% CI, R2=0.001, P<0.0001 and slope 0.00007 mGy/cm). But in this study, mean CTDIvol and SSDE values were low. There was negligible difference between mean CTDIvol and mean SSDE values. In our study, mean SSDE and CTDIvol values differed significantly. Our CTDIvol and SSDE values were higher compared to those obtained in the above mentioned studies. So, when scanner output is high, as in the case of diagnostic CT scans, SSDE varied significantly from CTDIvol. In such cases, SSDE may be a better estimate of patient dose. 

## Limitations:

 Sample population did not represent the entire range of body weight. There were very few patients weighing less than 30 kg and more than 100 kg. Therefore, we could not assess the impact of SSDE in estimation of doses in patients weighing on the extremes of the spectrum. We did not perform any phantom simulation studies to measure the actual dose. Therefore, we could not compare both the parameters with actual absorbed dose.

## Conclusion

 Absorbed dose from CT scans can be expressed by CTDIvol or Size specific dose estimates (SSDE). In systems with automatic exposure control, the dose delivered depends upon the body habitus of the patient. CTDIvol increases with increase in patient size. For all CTDIvol values, the corresponding SSDE values were found to be high. Since we did not perform phantom simulation studies, the actual dose could not be calculated. Our results suggest that the actual dose delivered may be higher than estimated by CTDIvol, especially in small-sized patients. 
